# Relationship between pickiness and subsequent development in body mass index and diet intake in obesity prone normal weight preschool children

**DOI:** 10.1371/journal.pone.0172772

**Published:** 2017-03-15

**Authors:** Jeanett Friis Rohde, Mina Nicole Händel, Maria Stougaard, Nanna Julie Olsen, Maria Trærup, Erik Lykke Mortensen, Berit Lilienthal Heitmann

**Affiliations:** 1 Research Unit for Dietary Studies at The Parker Institute, Bispebjerg and Frederiksberg University Hospital, The Capital Region, Frederiksberg, Denmark; 2 National Institute of Public health, University of Southern Denmark, Copenhagen, Denmark; 3 Department of Clinical Research, University of Southern Denmark, Odense Patient Data Explorative Network (OPEN), Odense University Hospital, Odense, Denmark; 4 Department of Public Health and Center for Healthy Aging, University of Copenhagen, Copenhagen, Denmark; 5 Boden Institute of Obesity, Nutrition Exercise & Eating Disorders, University of Sydney, Camperdown, New South Wales, Australia; 6 Department of Public health, Section for General Medicine, University of Copenhagen, Copenhagen, Denmark; University Sains Malaysia, MALAYSIA

## Abstract

**Background:**

Most children have periods in their life where they reject familiar as well as non-familiar food items and this is often referred to as pickiness. The consequences of pickiness may be malnutrition and, if prolonged, potentially lower body weight. However, studies investigating the consequence of pickiness on subsequent changes in diet intake and weight are limited.

**Objectives:**

To examine whether pickiness influences body mass index as well as diet intake over subsequent 15 months among obesity prone normal weight children aged 2–6 years.

**Methods:**

Data was obtained from the “Healthy Start” intervention study which included 271 children aged 2–6 years susceptible to overweight later in life. Information on pickiness was obtained from a parental questionnaire. Dietary habits were collected by 4-day dietary records filled in by the parents and height and weight were measured by trained health professionals and both measured twice over a 15 month period. Linear regression models were performed to assess the influence of pickiness on body mass index and diet with adjustments for possible confounders.

**Results:**

No differences in mean BMI Z-score were seen between picky/non-picky (P = 0.68) and little picky/non-picky (P = 0.68) children at 15 month follow-up. Picky children had a lower intake of protein (P = 0.01) than non-picky children despite no differences in total energy intake (P = 0.74), or in the other macronutrients, or the intake of fruit and vegetables, though children being a little picky had a lower intake of starch compared to non-picky children (P = 0.05). Results were essentially similar before and after adjustment for key covariates.

**Conclusion:**

Our study showed that BMI Z-score after 15 months follow-up was similar for picky and non-picky children. Picky children seemed to develop a lower protein intake despite similar total energy intake and diet composition.

## Introduction

Many children have periods in their life, where they reject familiar as well as non-familiar food items [1,2], and in particular the first few years of life seems a sensitive period in relation to developing food preferences [1,2]. Research suggests that by the age of 3 or 4 years, numerous children tend to develop a dislike for certain foods [3,4].There has been several attempts to characterize food-rejection, including food-neophobia, pickiness or fussiness [5]. Where food neophobia generally refers to the unwillingness to eat, or the unacceptance of new foods, 'pickiness/fussiness' often refers to the rejection of a large number of food items which may be familiar or unfamiliar to the child [5]. The short and long-term consequences of childhood pickiness has not been widely examined, but a review concluded that current diets of picky children often were low in fruit and vegetables and reflected little variety in food choices [5]. Further, research reports that picky children may also have a lower total energy intake than non-picky children [6]. Prospective studies examining pickiness and change in intake of diet and nutrients are generally lacking, and especially whether there is an association between pickiness and changes in macronutrient intake still needs to be clarified [6].

The adverse consequences in terms of malnutrition among picky eaters may, if prolonged, influence weight development of the child. Results from cross-sectional studies point to either a negative [7–10] or no association [11,12] between pickiness and anthropometric measures among children, though, no clear conclusions with regard to causal inference can be drawn from such studies. One previous longitudinal study showed that persistently picky children may be at higher risk of becoming underweight [13], but more longitudinal studies are needed to confirm these results.

Given this background, the objectives of the present study were to examine associations between pickiness in childhood and subsequent development in body mass index as well as diet intake over the subsequent 15 months.

## Materials and methods

This study was based on data collected over a 15 month period from children who participated in the randomised primary intervention study “Healthy Start” [14].

### Ethics

The Scientific Ethical Committee of the Capital Region in Denmark decided that according to Section 2.-(1) of the Danish Act on a Bioethics Committee System and the Processing of Bioethics Projects, the project was not a bioethics project and as a result did not need approval from the Danish Bioethics Committee (journal number H-A-2007-0019). Approval by The Danish Data Protection Agency regarding participant's data protection was obtained (journal number: 2015-41-3937). Written informed consent to use the collected data for research purposes was obtained from all parents of the participants.

### Healthy start intervention

The “Healthy Start” study was a randomized controlled intervention conducted between 2009 and 2011 in 11 municipalities around the greater Copenhagen area [14]. The aim was to prevent overweight in children aged 2–6 years, who were yet normal weight, but were susceptible to future overweight, as they were all either born with a high birth weight (> 4,000 grams), had a mother who was overweight prior to pregnancy (body mass index (BMI) > 28 kg/m^2^) and/or had a mother with low socioeconomic status (SES) (educational level ≤ 10 years) [14]. This intervention aimed to improve diet and physical activity habits, reduce stress and improve sleep quality and quantity. The methodology of the “Healthy Start” intervention study has been described in closer detail elsewhere [14].

### Study population

The “Healthy Start” intervention study initially invited 3,058 children (n: 1,523 intervention group and 1,535 control group) aged 2–6 years to participate and about 21% in each group agreed to participate [14]. Hence, the study population included a total of 543 normal weight children, 271 in the intervention group and 272 children in the control group. For the present study, children with missing information on the exposure variable (pickiness), the outcome variables (BMI or diet intake) or the possible confounders (group [intervention/control], age, sex and maternal education) were excluded, and therefore the final study population consisted of 271 children age 2–6 years.

### Measures

#### Pickiness

Parents were, at baseline, asked to complete a questionnaire which included questions on meal habits, and family well-being. Information on “*pickiness”* was self-reported by the parents and based on the question *“how would you describe your child’s way of eating*?*”* The parents could answer if they perceived their child as being “*picky*”, “*a little picky”* or “*likes everything*” (referred to as non-picky children).

#### Dietary measurements

Information on the child’s total energy- and macronutrient intake was obtained from a 4-day (Wednesday-Saturday) dietary record completed by parents at baseline and at follow-up, applying a picture book as guidance in reporting portion sizes [15]. The software Dankost 3000 was used for nutrition calculation (http://dankost.dk/). Dietary information was used to investigate if there were any differences in the macronutrient and fruit, vegetable and starch (rice, pasta and potatoes) intake between picky and non-picky children. Fruit was defined as: fresh, canned and frozen (excluding jam, fruit juice, dried fruit and fruit products with added sugar), vegetables as: fresh, canned and frozen (excluding fried onion, ketchup, pickles and potatoes) and starch as: rice, pasta or potatoes. All food groups were presented in g/day.

#### Anthropometric measurements

Data on the children’s height and weight were measured at baseline and follow-up by a trained health professional. Height was measured to the nearest 0.1 cm using a stature meter (Soehnle 5,002 or Charter ch200P). The children were in bare feet or in stockings. Body weight was measured to the nearest 0.1 kg using a mechanical weight or beam-scale type weight (TanitaBWB-800 or SV-SECA 710). Children were measured in underwear and were asked to urinate before weighing. If the children were using a diaper, a new one was put on before weighing. BMI z-scores were generated using the Lambda-Mu-Sigma method, which summarizes the changing distributions of the dependent variable by the median, the coefficient of variation and skew expressed as Box-Cox power [16]. A power transformation in increments of 0.1 years was used, applying national reference z-scores to the study population [17].

#### Confounders

Possible confounders were selected *a priori* based on the existing literature [1,18,19–22]. Information on these variables was obtained from the parental questionnaire completed by the parents at baseline and from The Danish Medical Birth Registry. Information on maternal education level was obtained from the parental questionnaire, and was collapsed into three groups: “No academic training”, “Academic training for up 3–4 years” and “University degree”. Information on the children’s age and sex was obtained from The Danish Medical Birth Registry. Since information for this study was based on data from the “Healthy Start” intervention we further adjusted for the intervention status (intervention vs. control group).

### Statistical methods

Linear regression models were used to examine the influence of pickiness on BMI, total energy intake, macronutrients, fruit, vegetables and starch with adjustments for baseline measure of outcome, group (intervention/control), age, sex and maternal education. Potential interactions between pickiness and intervention/control group were explored for all outcomes by adding a product term to the models. Significant interactions were further evaluated through stratified analyses. Moreover, analyses of non-completers were performed to investigate if completers differed from non-completers with regard to pickiness status, BMI, total energy intake, age, sex, group and maternal education level. Differences were considered statistically significant when P<0.05. All statistical analyses were performed using Intercooled version Stata 14.0 (StataCorp LP, College Station, Texas; www.stata.com).

## Results

Table 1 shows baseline characteristics of the participating children. In total 16% of the parents reported their child to be picky, 42% a little picky, and 42% as non-picky. Significant differences in age were observed between children being a little picky, picky or non-picky (P = 0.03) (Table 1). No differences were observed between picky, little picky and non-picky children in relation to baseline BMI Z-score, total energy intake, sex, maternal education and group (intervention/control) (Table 1). Analyses of non-completers showed a higher total energy intake among the participating children compared to non-completing children. Moreover, a higher participating rate was seen for children from the control group. No differences were observed in relation to pickiness status, sex, age, BMI Z-score and maternal education (S1 File).

**Table 1:** Baseline characteristics of the included children stratified by pickiness status. The difference between groups was tested using oneway anova for continuous variables and Chi-squared test for categorical variables. Results are presented as median (5–95 percentiles) unless otherwise stated^1^.

	Non-picky	Little picky	Picky	
	Median *(5;95 percentiles)*	Median *(5;95 percentiles)*	Median *(5;95 percentiles)*	P-value
**Age (yrs)**	3.9	3.9	4.5	0.03
	*(2*.*3;5*.*8)*	(2.7;5.6)	*(2*.*6;5*.*7)*	
**BMI Z-score (SD)**	0.1	0.2	0.1	0.12
	*(-1*.*2;1*.*1)*	(-1.0; 1.3)	*(-1*.*3;1*.*2)*	
**Total energy intake (MJ)**	4.7	4.9	5.0	0.39
	*(3*.*5; 6*.*7)*	(3.3; 6.5)	*(3*.*1; 6*.*5)*	
	**Percent**	**Percent**	**Percent**	
**Boys**	57	61	54	
**Girls**	43	39	46	0.66
**Maternal Education**				
No academic training	21	23	19	
Academic training for up 3–4 years	57	46	67	
University degree	22	31	14	0.11
**Group**				
Intervention	40	50	47	
Control	60	50	53	0.33

^1^ Number of participants: Non-picky (42%; n = 113), Little Picky (42%, n = 115) and picky children (16%; n = 43)

Pickiness was not associated with development in BMI Z-score after the 15 month (β: 0.03, 95%CI: -0.13; 0.19, P = 0.68) (Table 2). The same pattern of no association after 15 month of follow up was seen for children being a little picky in relation to BMI Z-score (β: 0.03, 95%CI -0.10; 0.16, P = 0.68). The results were essentially similar before and after adjustment for covariates (Table 2). A lower protein intake was observed after 15 months of follow up among the picky children compared to non-picky children (β: -1.16, 95%CI: -1.99; -0.33, P = 0.01), also after adjustment for covariates (β: -1.17, 95%CI: -2.02; -0.32, P = 0.01). In addition, a lower starch intake was observed after 15 months of follow up among the little picky children compared to the non-picky children (β: -9.30, 95%CI: -18.43; -0.12, P = 0.05). This association remained significant after adjustments (β: -9.36, 95%CI: -18.94; -0.19, P = 0.05). However, pickiness was not associated with development over 15 months in total energy intake, neither before nor after adjustment for potential confounders (Table 2). Moreover, no differences related to pickiness were observed for the development over 15 months in the intakes of fat, carbohydrates, added sugar, fruit, vegetables or starch intake between picky and non-picky children. Finally, no associations were observed between being a little picky and developments in intakes of protein, fat, carbohydrates, added sugar, fruit and vegetables compared to non-picky children (Table 2).

**Table 2:** Influence of pickiness on BMI z-score, total energy intake, macronutrients and fruit, vegetables and starch intake after 15 month follow up (n = 271)^1^.

	Crude model^2^			Adjusted model^3^		
	β	95% CI	P-value	β	95% CI	P-value
**BMI Z-score**						
*Picky eaters*	0.03	-0.13; 0.19	0.68	0.04	-0.13; 0.21	0.63
*A little picky*	0.03	-0.10; 0.16	0.68	0.03	-0.10; 0.16	0.65
**Total energy intake (kJ)**						
*Picky eaters*	-62.69	-436.84; 311.46	0.74	-121.34	-487.82; 245.14	0.52
*A little picky*	163.16	-111.50; 437.83	0.24	135.14	-135.16; 405.43	0.33
**Protein (E%)**						
*Picky eaters*	-1.16	-1.99; -0.33	0.01	-1.17	-2.02; -0.32	0.01
*A little picky*	-0.36	-0.98; 0.25	0.25	-0.37	-1.01; 0.26	0.25
**Fat (E%)**						
*Picky eaters*	0.42	-1.36; 1.76	0.37	0.29	-1.50; 2.09	0.75
*A little picky*	0.55	-0.66; 1.76	0.37	0.54	-0.67; 1.76	0.38
**Carbohydrates (E%)**						
*Picky eaters*	0.58	-1.30; 2.46	0.54	0.72	-1.20; 2.63	0.46
*A little picky*	-0.22	-1.52; 1.08	0.74	-0.19	-1.52; 1.15	0.78
**Added sugar (E%)**						
*Picky eaters*	1.07	-4.06; 6.20	0.85	-0.45	-5.53; 4.64	0.86
*A little picky*	0.38	-3.68; 4.45	0.85	0.26	-3.64; 4.16	0.90
**Fruit (g/day)**						
*Picky eaters*	-1.48	-21.94; 18.97	0.89	2.92	-18.13; 23.97	0.79
*A little picky*	-4.09	-19.66; 11.48	0.61	-3.77	-19.23; 11.70	0.63
**Vegetables (g/day)**						
*Picky eaters*	-1.62	-27.10; 23.85	0.90	0.24	-24.55; 25.02	0.99
*A little picky*	-1.46	-18.14; 15.22	0.86	-2.24	-19.49; 15.01	0.80
**Starch (g/day)**						
*Picky eaters*	-9.89	-22.58; 2.80	0.13	-10.16	-23.25; 2.93	0.13
*A little picky*	-9.30	-18.43; -0.17	0.05	-9.36	-18.94; -0.19	0.05

^1^ Standard errors of the parameter estimates where calculated by bootstrapping. Reference group: non-picky children.

^2^ Crude model: Pickiness and BMI or each given dietary outcome adjusted for baseline intake and group (intervention/control).

^3^ Adjusted model Pickiness and BMI or each given dietary outcome adjusted for baseline intake and group (intervention/control), sex, age and maternal education.

We found no differences between the intervention and the control group in the relations between pickiness and BMI Z-score or intakes of total energy, protein, fat and carbohydrate after the 15 months of follow-up.

## Discussion

In the present study we followed the children for 15 months and found no indication that pickiness was influencing development in BMI Z-score during this period. Most previous studies were cross sectional, but generally showed that pickiness was related to current underweight and lower BMI [7,8,13,23]. Authors from these studies have suggested that that if prolonged, this may influence growth and weight development of the child [7]. However, few previous studies have examined this over time, and those that have, generally did not find an association between selective eating among children and weight development [11,24,25] which is in accordance with our finding.

Also, we did not find differences in development over 15 months in total energy intake, intake of macronutrients, or fruit and vegetables between picky and non-picky children, but picky children seemed to have developed a lower intake of protein than non-picky children. Also, children being a little picky had developed a lower intake of starch (rice, pasta and potatoes) compared to non-picky children. To our knowledge other studies examining associations between pickiness and the development in diet intake over time are absent from the current literature [6], and we can therefore not directly compare our results to those of other similar studies. However, our findings are supported by one previous retrospective population-based cohort study showing no significant differences in total energy between picky and non-picky eaters at 14 month of age [26], but in disagreement with another cross sectional study that found a lower energy intake among picky than non-picky children [21]. Also, previous studies have found that picky children seems to have less variety in their diet, and that the diets of picky children often are low in fruit and vegetables and in protein-rich foods [5,6], which may compromise intake of certain nutrients [6]. These results are in support of our findings of development of lower protein and starch intakes among picky and little picky children after the 15 months of follow up. Also, despite not being significant, trends were generally in favor of developing a lower intake of fruit and vegetables and higher intake of added sugar among picky/little picky children compared to non-picky children. This indicates that compared to non-picky children, the picky children may develop diets with higher intake of refined products such as dried fruit, candy and sugar-sweet beverages and hence potentially a poorer micronutrients composition.

The strengths of our study include the prospective design and that height and weights were measured objectively by health consultants rather than being self-reported, which mitigates reporting bias. There are also a number of limitations to the study, such as a limited sample size, and the use of simple questions to measure pickiness rather than multi-item validated scales. Indeed, although the parents stated that they found their child to be picky, differences in how parents define pickiness may exist. This potential misclassification introduced using this more crude information may potentially have led to attenuation of our observed associations.

The generalizability of the results is subject to some limitations. Our study population was composed of normal weight children that were all in the risk of becoming overweight later in life, based on three risk factors used as inclusion criteria in the “Healthy Start” study: a high birth weight (> 4,000 grams), maternal pre-pregnancy BMI > 28 kg/m^2^ and/or maternal educational level ≤ 10 years, and hence, our results may not apply to normal weight children without a predisposition to obesity. One could argue that picky eating in obesity-prone children might result in normal weight as opposed to underweight, for example, and be interpreted as no association. Also, the children participating were all Danish Caucasians and from a society with generally high affluence. Hence our results may not be generalized to children from less affluent societies or other cultures.

Our study is among the first to examine the nutritional consequences of pickiness over time among preschool children, and the results implies that even though no difference were observed in total energy intake between picky and non-picky children the diets that picky children develops may be of lower micronutrient quality than non-picky children, referring to a lower intake of fruit and vegetables and a higher intake of fat and added sugar, although further studies are needed to confirm our findings. Furthermore, studies that explore what potentially causes pickiness are needed. One research area of particular interest would be the atmosphere during the family meal, since mealtimes in general seem to be the setting with the greatest potential to influence diet intake of young children [1,3,19].

## Conclusion

Overall our results suggest that among young Danish children pickiness may not be related to the development in BMI, but may influence the development in diet by leading to a lower protein intake. Thus, picky children may develop a lower diet quality than non-picky children. Our results should be generalized with caution only, because of the selected study population, and because further studies are needed to confirm these findings.

## Supporting information

S1 File Distribution of sex, pickiness status, group, maternal education, age, BMI z-score and total energy intake among completers and non-completers. (DOCX)
