# Supplementary material for: Relationship between pickiness and subsequent development in body mass index and diet intake in obesity prone normal weight preschool children
Source: PLoS One. 2017 Mar 15;12(3):e0172772. doi: 10.1371/journal.pone.0172772 (PMC5351873; doi:10.1371/journal.pone.0172772)
Supplement: S1 File — (DOCX) [file pone.0172772.s001.docx]

| **S1 file: Distribution of sex, pickiness status, group, maternal education, age, BMI Z-score and total energy intake among completers and non-completers.** | | | | |
| --- | --- | --- | --- | --- |
|  | **Completer** | **Non-completers** | **p-value** |  |
|  | **%** | **%** |  |  |
| **Sex** |  |  |  |  |
| Boy | *58* | *58* | *0.90* |  |
| Girl | *42* | *42* |  |  |
|  |  |  |  |  |
| **Pickiness** |  |  |  |  |
| Non-picky | *42* | *40* |  |  |
| Little picky | *42* | *44* |  |  |
| Picky | *16* | *16* | *0.92* |  |
|  |  |  |  |  |
| **Maternal education** |  |  |  |  |
| No academic training | *21* | *29* |  |  |
| Academic training for up 3-4 years | *54* | *47* |  |  |
| University degree | *25* | *24* | *0.11* |  |
|  |  |  |  |  |
| **Group** |  |  |  |  |
| Intervention | *45* | *55* |  |  |
| Control | *55* | *45* | *0.02* |  |
|  |  |  |  |  |
|  | **Mean** | **Mean** |  |  |
| **Age (yrs)** | *4.0* | *4.0* | *0.70* |  |
|  |  |  |  |  |
| **BMI Z-score (SD)** | *0.1* | *0.1* | *0.70* |  |
|  |  |  |  |  |
| **Total energy intake (MJ)** | *4.9* | *4.6* | *>0.01* |  |
|  | | | | |
